# Design and Simulation Analysis of a Temperature Control System for Real-Time Quantitative PCR Instruments Based on Key Hot Air Circulation and Temperature Field Regulation Technologies

**DOI:** 10.3390/mi17020169

**Published:** 2026-01-28

**Authors:** Zhe Wang, Yue Zhao, Yan Wang, Chunxiang Shi, Zizhao Zhao, Qimeng Chen, Lemin Shi, Xiangkai Meng, Hao Zhang, Yuanhua Yu

**Affiliations:** 1 School of Life Science and Technology, Changchun University of Science and Technology, Changchun 130022, China; 2 Jilin Provincial Institute of Metrology Science, Changchun 130103, China

**Keywords:** PCR instrument, airflow rectification system, cruciform frame temperature control system, reliability

## Abstract

To address the technical bottlenecks commonly encountered with real-time quantitative PCR instruments, such as insufficient ramp rates and uneven chamber temperature distribution, this study proposes an innovative design scheme for a temperature control system that incorporates key hot air circulation and temperature field regulation technologies. By combining the PCR instruments’ working principles and structural characteristics, the failure mechanisms associated with the temperature control system are systematically analyzed, and a reliability-oriented thermodynamic analysis model is constructed to clarify the functional positioning of core components and to systematically test the airflow uniformity, temperature dynamics, and nucleic acid amplification efficiency. An integrated fixture for airflow rectifier and cruciform frames is designed, which enables precise quantitative characterization of the system temperature uniformity, ramp rates, and amplification efficiency on a multi-condition comparison platform. Through modeling analysis combined with experimental validation, the thermal performance differences among various heating chamber structures are compared, leading to a multidimensional optimization of the temperature control system. The test results demonstrate outstanding core performance metrics for the optimized system: the up ramp reaches 7.5 ± 0.1 °C/s, the down ramp reaches 13.5 ± 0.1 °C/s, and the steady-state temperature deviation is only ±0.1 °C. The total duration for 35 PCR cycles is recorded at 16.3 ± 0.6 min, with a nucleic acid amplification efficiency of 98.9 ± 0.2%. The core performance metrics comprehensively surpass those of mainstream global counterparts. The developed temperature control system is well-suited for practical applications such as rapid detection, providing critical technological support for the iterative upgrade of nucleic acid amplification techniques while laying a solid foundation for the engineering development of high-performance PCR instruments.

## 1. Introduction

In today’s fast-paced technological landscape, gene technology has emerged as a research hotspot and core application across multiple domains. Polymerase Chain Reaction (PCR), a pivotal technology in genetic engineering, enables the rapid in vitro amplification of deoxyribonucleic acid (DNA) fragments [1]. Its applications span environmental monitoring, forensic investigation, species identification, food safety inspection, agricultural breeding, and clinical research [2]. PCR, recognized for its ease of operation, high sensitivity, strong specificity, and exceptional amplification efficiency, is also referred to as cell-free molecular cloning or in vitro primer-directed enzymatic amplification. The standard PCR procedure comprises four stages, pre-denaturation, denaturation, annealing, and extension, all of which rely on precise temperature control provided by PCR instruments [3]. On the technological level, PCR has evolved into a collaborative landscape involving conventional qualitative PCR, real-time quantitative PCR (qPCR), digital PCR (dPCR), and isothermal amplification techniques. Traditional PCR technology, characterized by maturity and stability, offers advantages such as user-friendly operation and controllable cost, making it a mainstream tool for fundamental research and routine pathogen screening. However, its lack of quantitative capability limits its application in precision diagnostics [4,5]. By integrating fluorescence detection modules, qPCR enables real-time monitoring and quantitative analysis of the amplification process, establishing itself as a core technology for clinical quantitative testing [6]. dPCR technology achieves breakthroughs through its absolute single-molecule quantitative advantage. It is classified into droplet-based (ddPCR) and chip-based (cdPCR) types. ddPCR can generate millions of reaction droplets, but faces challenges such as droplet fusion during thermal cycling and dependence on expensive supporting equipment. cdPCR forms stable reaction units through physical partitioning. While offering lower cost and simpler operation, cdPCR imposes stringent operational requirements. Manual operation of chip-based (cdPCR) systems may introduce issues such as air bubbles and uneven droplet volume, which directly affect quantitative accuracy [7]. Nevertheless, the currently available commercial PCR instruments display common shortcomings: most devices suffer from lengthy thermal cycling duration, some products fail to maintain constant-temperature stability, rapid-type instruments often lack sufficient sensitivity, while another category features bulky design, complex operation, and high costs, rendering them unsuitable for diverse practical applications [8,9,10]. These limitations hinder the deployment of some PCR instruments in resource-constrained scenarios such as point-of-care testing and on-site environmental monitoring.

Currently, the classification systems for PCR instruments remain inconsistent. Influenced by researchers’ differing technical focuses and application contexts, Microfluidic PCR has garnered significant attention in recent years. Microfluidic PCR integrates microfluidic technology with conventional PCR, utilizing micro-scale channels to miniaturize reaction volumes. This approach reduces reagent consumption, enhances heat transfer efficiency, and facilitates integration with upstream sample preparation and downstream detection modules. These inherent advantages make microfluidic PCR an ideal technological solution for high-throughput analysis, rapid point-of-care testing, and portable diagnostic devices. Based on their thermal cycling mechanisms, microfluidic PCR systems are primarily categorized into three types: stationary-chamber microfluidic PCR, flow-through PCR, and convective PCR [11]. Stationary-chamber microfluidic PCR places the reaction mixture within a fixed microchamber and achieves amplification through cyclical temperature changes. Although its principle is similar to traditional PCR, the reduced sample volume significantly improves its thermal response speed [12]. Flow-through PCR drives the reaction solution through microchannels across different constant-temperature zones to accomplish thermal cycling, enabling continuous rapid amplification. Convective PCR relies on natural or forced thermal convection to establish a temperature gradient within the reaction chamber, which eliminates the need for complex temperature cycling mechanisms, and facilitates device miniaturization [13]. Domestic entities have achieved phased breakthroughs in this field: regarding the convective type, the portable cPCR amplifier developed by Luoyang Pulike Biotechnology Co., Ltd.located in Luoyang City, Henan Province, China. completes nucleic acid amplification by inducing solution convection, which considerably shortens the detection time compared to conventional devices, thereby enabling portable point-of-care diagnostics. However, its solution temperature stability requires further improvement. In the realm of stationary-chamber microfluidic PCR, companies such as MGI Tech and Bioer Technology have attained technological autonomy. Their products meet primary medical care and research demands, with annual production capacity reaching 300–500 units per line. The core Peltier heating technology ensures detection sensitivity, although the ramp rates require optimization. Meanwhile, flow-through technology emphasizes integration with microfluidic technology, yet encounters the same bottlenecks as its international counterparts: the channel surfaces tend to adsorb biomolecules, and their reliance on expensive external pumps hinders precise quantitative detection. Foreign countries hold a more mature technological presence in the fields of stationary-chamber microfluidic PCR and flow-through PCR instruments. Among the stationary-chamber products, Bio-Rad’s CFX96 real-time PCR system achieves nucleic acid quantification through precise temperature control and efficient fluorescence detection modules, while the T100 thermal cycler from the same series enables flexible temperature gradient settings, thus facilitating experimental condition optimization and efficiency enhancement [14]. Flow-through technology achieves amplification by circulating the reaction solution through distinct constant-temperature zones within microchannels. However, the channel surfaces readily adsorb biomolecules, inhibiting the PCR and leading to reduced sensitivity. Moreover, the need for expensive external pumps complicates precise quantitative detection [15,16]. Convective heating technology similarly fails to resolve constant-temperature stability issues, with challenges such as channel adsorption and quantification difficulties persisting [17].

Temperature control, as a core factor determining PCR performance, directly influences amplification efficiency, specificity, and repeatability. Consequently, numerous temperature control methods suitable for PCR and microfluidic PCR systems have been developed, each possessing distinct advantages and limitations [18]. Common heating techniques include Peltier element heating, infrared heating, plasma-laser heating, and hot-air circulation heating. Peltier elements are widely employed in commercial PCR instruments due to their low cost, compact size, and ability to provide both heating and cooling functions [19]. However, Peltier-based systems suffer from issues such as slow temperature response and uneven temperature distribution within the reaction chamber, which become particularly pronounced in large-volume or multi-channel configurations [20]. Infrared heating and plasma-laser heating technologies can significantly increase heating rates, enabling rapid thermal cycling. They are hindered by drawbacks including low cooling efficiency, high energy consumption, and reliance on expensive optical components, limiting their commercial adoption. Hot-air circulation-based temperature control achieves uniform temperature distribution through forced convection. In recent years, it has gradually emerged as a potential solution for improving temperature uniformity and shortening thermal cycling times, though its application in PCR systems remains in the early stages [21].

Despite the worldwide development of various real-time quantitative PCR instruments, practical engineering applications reveal that none fulfill the dual requirements of high-efficiency, rapid amplification alongside uniform, precise temperature control within the reaction chambers [22]. Hence, this study focuses on developing a temperature control system for the quantitative PCR instruments based on hot air circulation and temperature field regulation. Initially, the system structure design was completed, employing cruciform frames and turbulent fans, along with an additional rectifier to enhance the temperature control performance. Next, material selection was optimized to improve the thermal conductivity, and the dimensional parameters and assembly positions of each component were specified. After determining the final instrument structure through modeling analysis and simulation testing, comprehensive performance verification was conducted. The final product features strong anti-interference capability, excellent robustness, high measurement accuracy, compact size, and controllable cost, which enables efficient, rapid, and stable temperature cycling, thus effectively addressing the shortcomings of existing instruments.

## 2. Technical Solution and Structural Layout of the Temperature Control System

Based on key technologies of hot air circulation and temperature field regulation, the temperature control system for real-time quantitative PCR instruments developed in this study achieves breakthroughs in core performance and engineering implementation, which rely heavily on the deep support of micro-mechatronics technology in terms of precision manufacturing, integrated design, and rapid response characteristics. The intrinsic connection between the two is indirectly corroborated through the system’s structure, the functionality of its core components, and its performance metrics. The temperature control system adopted a compact nested structure of heating cylinder, cruciform frame, and reaction chamber. The feasibility of this spatially optimized design depends on the high integration of miniature actuation elements and precision structures. This is paired with a miniature servo motor that enables millisecond-level speed control. Its small footprint perfectly adapts to the narrow-gap flow channels within the cruciform frame. The precise driving capability of the servo motor provides the hardware foundation for dynamic regulation of the hot air circulation intensity, fully leveraging the advantages of small size and high precision inherent to micro-mechatronics technology.

The distributed array of miniature PT100 temperature sensors and hot-film anemometers arranged along the inner walls of the flow channels ensures the accuracy of temperature field regulation. With nanosecond-level response speed and micrometer-level detection resolution, these sensors enable real-time acquisition of multi-point temperature and airflow velocity within the chamber. This provides high-fidelity data support for the closed-loop temperature control algorithm, directly contributing to the system’s achievement of its core performance indicators: a heating rate of 7.5 ± 0.1 °C/s, a cooling rate of 13.5 ± 0.1 °C/s, and temperature uniformity of ±0.1 °C. The processing and packaging techniques of these miniature sensing elements, along with designs for interference resistance and stability, guarantee the reliability of detection data during rapid thermal cycling. This represents a direct manifestation of the deep coupling between the temperature control system and micro-mechatronics technology at the component level. The cruciform frame serves as the core heat transfer structure of the temperature control system. Its design, featuring channels that are wider at the top and narrower at the bottom, along with optimized asymmetric angle parameters through fluid dynamics simulation, achieves simultaneous enhancement of uniform airflow distribution and heat exchange efficiency. The precision manufacturing of this frame relies on high-precision milling and surface treatment technologies within micro-machining processes. The dimensional deviation of its tooth-shaped groove structure is controlled within ±0.05 mm, ensuring uniformity in heating wire winding and consistency in heat transfer pathways. This capability for structural machining with micron-level precision is a core advantage of micro-mechatronics technology in the field of precision manufacturing. Furthermore, the preparation of the microfluidic chips compatible with the system, featuring nanoliter-scale reaction chambers and micrometer-scale channels, also relies on MEMS photolithography and etching technologies. The precise compatibility between the chip and the temperature control system highlights the advantages of miniaturized reaction carriers and demonstrates the deep adaptation of the temperature control system’s design to the application scenarios of micro-mechatronics technology.

From the perspective of system performance iteration, the temperature control system achieves portable design and efficient temperature control. In essence, this is an extension of the characteristics of micro-mechatronics technology: “high integration, low power consumption, and rapid response”. The integrated application of miniature actuation and sensing elements can significantly reduce the system’s dead volume and thermal load. Specifically, the total time for 35 PCR cycles is shortened to 16.3 ± 0.6 min. The stringent demand of the temperature control system for spatiotemporal resolution of the temperature field has, in turn, driven the optimization and upgrading of micro-mechatronics technology in areas such as interference resistance and stability.

### 2.1. Research Framework for the Temperature Control System

**Figure 1:**
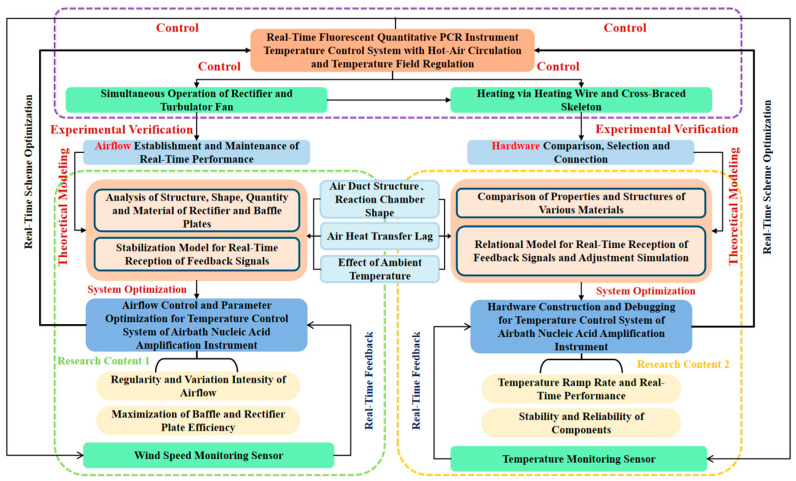
Research block diagram.

The accuracy of nucleic acid amplification relies on the stable support provided by the temperature control system. For real-time quantitative PCR instruments employing hot air circulation and temperature field regulation, the primary challenges in temperature control include airflow uniformity and hardware response speed. Hence, the research framework for the temperature control system of these PCR instruments, which is advanced through a closed-loop logic involving theoretical modeling, system optimization, experimental validation, and real-time feedback, consists of two core areas: Airflow Control and Parameter Optimization; and Hardware Assembly and Debugging. Figure 1 illustrates the system research framework.

The Airflow Control and Parameter Optimization began with theoretical modeling, which involved analyzing the structure, shape, and materials of the rectifier and baffle plates to construct a stable model capable of receiving real-time feedback signals, where factors such as duct structure, reaction chamber geometry, and air heat transfer lag were considered. Following the model-based system optimization, experimental validation was conducted to establish airflow and maintain its real-time stability. Airflow control was achieved through coordinated operation of rectifier and turbulator fan. Ultimately, the feedback data from wind speed sensors was utilized to optimize the airflow regularity and enhance the efficiencies of the rectifier and baffle plates.

The Hardware Assembly and Debugging also relied on theoretical modeling, through which the material properties and structures were compared to construct a relational model that adjusts simulations based on feedback signals. Following the system optimization, experimental validation was conducted for hardware selection and connectivity. Heating functionality was achieved using heating wires and cruciform frames, then the temperature sensors provided feedback data for the optimization of ramp rates, as well as device stability and reliability.

### 2.2. Technical Solution for the Temperature Control System

Leveraging steady advancements in microelectronics, high-precision sensor technology, and precision manufacturing, the temperature control systems for real-time quantitative PCR instruments employing hot air circulation and temperature field regulation are iteratively upgrading toward higher precision, faster response, and greater stability [23]. This study focuses on the stringent temperature requirements during the denaturation, annealing, and extension phases of nucleic acid amplification reactions. By coordinating a rectifier with a heating cylinder and incorporating a turbulator fan to create a forced convection module, the reaction chambers were placed within a controllable air thermal field for achieving dynamic temperature regulation [24]. The temperature field uniformity, along with the ramp rates, were determined by capturing the real-time temperature signals from high-precision sensors embedded within the chambers and analyzing the chamber airflow circulation patterns. This allows for precise temperature control during nucleic acid amplification, thus ensuring the specificity of amplification products and the reliability of detection results [25].

**Figure 2:**
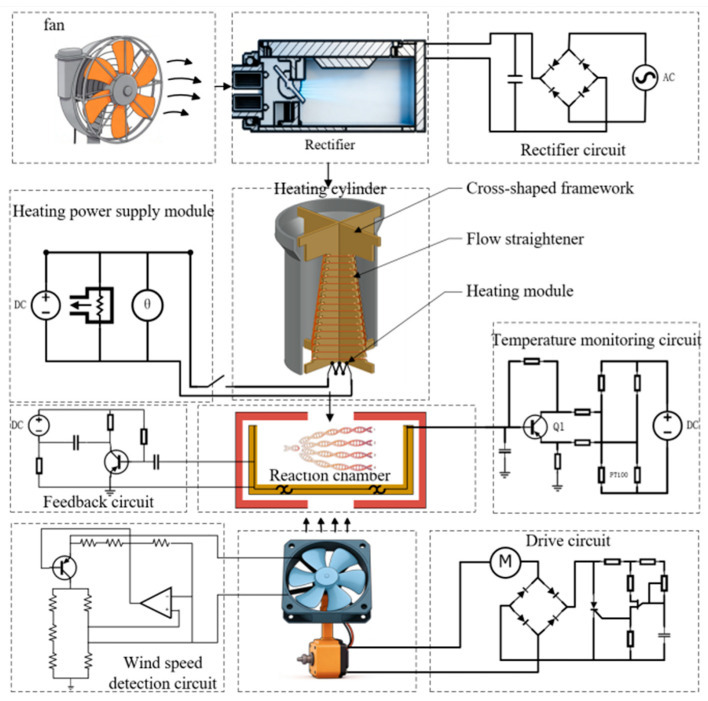
Working principle diagram.

The specific working principle is depicted in Figure 2. The system takes alternating current (AC) as the primary input and direct current (DC) to power the low-voltage circuits. Motor (M) drives the monitoring fan to regulate the airflow in the reaction chamber, while the switching element (Q1) adjusts the heating power based on temperature deviations to realize closed-loop temperature control.

### 2.3. Structural Layout of the Temperature Control System

**Figure 3:**
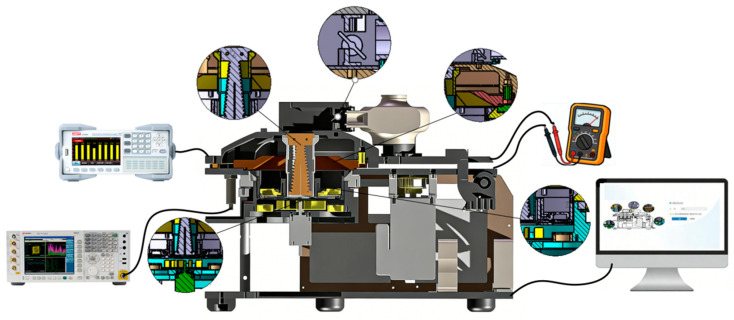
Schematic of the hot air circulation-based temperature control system.

The temperature control system designed for real-time quantitative PCR instruments, which employs hot air circulation and temperature field regulation, operates under the precise control signals generated by the preset temperature program, enabling the rectifier and heating cylinder to work in coordination within the directed airflow channels created by the turbulator fan. Consequently, the reaction chamber stability within a dynamic temperature field is guaranteed while achieving efficient temperature regulation [26]. Utilizing high-precision temperature sensors embedded within the chambers, the temperature control system continuously tracks and monitors the dynamic temperature variations in the core reaction zone. Such nonlinear response and peak phenomenon result from the combined effects of varying temperature requirements across different stages of nucleic acid amplification, as well as the thermal inertia inherent to the chambers [27]. After indirectly regulating the temperature stability of the reaction system through this mechanism, the corresponding regulation patterns are fed back in real time via the temperature sensors to the control module, thereby ensuring the specificity and efficiency of the nucleic acid amplification reactions [28]. A schematic diagram of the hot air circulation-based temperature control system for real-time quantitative PCR is displayed in Figure 3.

The structural design of the temperature control system, which employs hot air circulation and temperature field regulation, is particularly crucial for the entire PCR instruments to achieve precise temperature control during the nucleic acid amplification reactions and to ensure the specificity and reliability of detection results [29]. Considering the system’s application in medical diagnostics and life science research, it must accommodate rapid parallel testing of multiple samples while meeting the high-frequency usage demands, and avoid temperature inhomogeneity and slow cooling within the nucleic acid reaction chambers, which could lead to reduced amplification specificity, detection result deviations, and inefficient testing. Thus, the system demands exceptional structural stability and responsiveness, while aiming to simplify the routine device transportation and reduce maintenance costs. A dedicated temperature control system structure was designed for the real-time quantitative PCR instruments, which features hot air circulation and temperature field regulation. This system incorporates components such as a rectifier, heating cylinder, and turbulator fan, all designed with optimized material selection and structural integrity.

### 2.4. Overall Structure and Microfluidic Chip Layout

**Figure 4:**
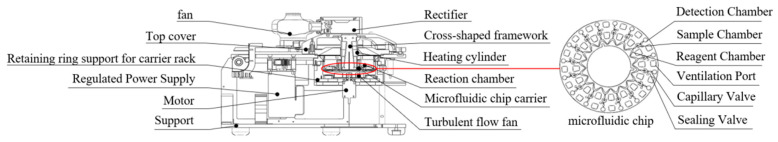
Overall and microfluidic chip structure diagram.

The overall structure of this real-time fluorescent quantitative PCR instrument adopts a compact nested design concept. Each module is highly integrated around the core temperature control unit, which combines the heating cylinder, cruciform frame, and reaction chamber, meeting the portability requirements for point-of-care testing scenarios. As the core interface unit between the temperature control system and the detection objects, the reaction carrier adaptation module incorporates a precision positioning structure, allowing flexible compatibility with mainstream reaction carriers such as microfluidic chips and 96-well plates. The microfluidic chip, characterized by its miniaturization and high adaptability, serves as the core reaction carrier in this study. The microfluidic chip is embedded within the central area of the reaction chamber, positioned directly below the airflow outlet of the cruciform frame. It forms a rigid connection with the temperature control system via a three-point positioning snap-fit, ensuring that the hot air circulation uniformly acts upon the chip surface and providing a structural foundation for precise temperature field regulation and rapid response. This overall layout ensures the functional independence of each module and miniaturized integration through spatial hierarchy optimization, embodying a design logic that maximizes integration density and spatial utilization. Figure 4 illustrates the overall structure and the microfluidic chip layout.

The microfluidic chip employs a functionally partitioned, integrated layout design. Its overall dimensions are compact, with a radius of merely 45 mm and a thickness of 1.8 mm. The chip orderly integrates three main functional areas, the reagent zone, the sample zone, and the detection chamber, which are precisely interconnected via a micron-scale channel network. The sample loading zone features 16 independent inlets, corresponding to a 16-channel parallel layout in the reaction zone. Each channel is equipped with an independent micro-reaction chamber and features a serpentine design to extend the reaction path and ensure uniform fluid distribution. The detection chamber is positioned directly above the sample zone and is designed with optical transparency to match the signal acquisition requirements of the fluorescence detection module. This layout achieves high-throughput detection capability and minimizes the chip footprint through the compact arrangement of functional zones. The positional error of each functional zone is controlled within ±0.05 mm, fully reflecting the core characteristics of miniaturization and high-density integration inherent to microfluidic chips.

From a structural perspective, the microfluidic chip uses optically transparent glass as its substrate material. It is fabricated using micro-electro-mechanical system (MEMS) processing techniques, including photolithography and dry etching. The junctions between channels and chambers incorporate smooth transition designs, effectively reducing fluidic resistance and dead volume. The chip surface is covered with an ultra-thin sealing layer, which ensures the sealing integrity of the reaction system and reduces the thermal transfer path length. The bottom layer is bonded to a thermally conductive substrate via plasma bonding technology, enhancing the thermal coupling effect with the temperature control system. This micron-scale structural design is a typical application of micro-mechanical precision machining technology, with channel dimensions and chamber volumes falling within the core operational range of micro-mechanical techniques. The sub-micron level fabrication accuracy achieved through MEMS processes ensures structural consistency across all channels, providing a structural guarantee for the synchronization of temperature responses in different reaction units.

The miniaturization characteristics of this microfluidic chip are manifested not only in its physical dimensions and structural scale but also in the functional optimization achieved through deep integration with micro-mechanical technology. The design of micron-scale channels and nanoliter reaction chambers reduces sample and reagent consumption by over 80% compared to traditional 96-well plates. Simultaneously, by significantly shortening the thermal transfer distance, the reaction system can respond rapidly to the temperature ramp commands from the control system. The synergistic effect between the microfluidic chip and the hot air circulation technology further enhances the thermal response speed of the temperature control system. The high integration density and miniaturization of the chip are realized precisely by leveraging the advantages of micro-mechanical technology in precision manufacturing and multi-component integration. This coupling of structure and technology highlights the core features of the microfluidic chip, including miniaturization, integration, and efficiency, which provides critical support for the temperature control system to achieve high-precision and rapid-response thermal performance.

## 3. Reliability Analysis of the Temperature Control System

### 3.1. Temperature Control System

For the temperature control system of the PCR instruments employing hot air circulation and temperature field regulation, its structural design is central to achieving the precise temperature control during nucleic acid amplification reactions, which ensures the detection result specificity, and helps maintain the detection efficiency. In the course of system structural design, the temperature control uniformity, stability, and efficiency must be key considerations. Through a multidimensional comparative analysis of existing mainstream material choices and design schemes, this study ultimately adopts a core hardware architecture comprising heating wires, heating cylinder, cruciform frames, fans, and servo motor for the temperature control system.

**Figure 5:**
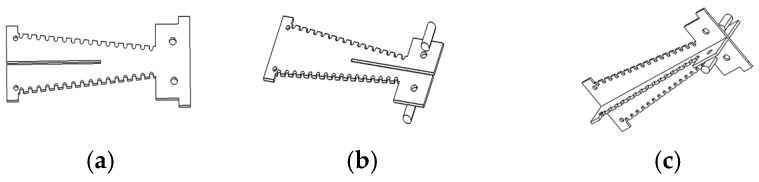
Structural schematic of the cruciform frames. (**a**) Cruciform frame 1. (**b**) Cruciform frame 2. (**c**) Complete frame assembly.

The cruciform frame structure used in this study consists of two interlocking trapezoidal frames with serrated edges. Frame 1 features a top resembling a thin rectangular plate, with two small rectangles directly attached to its upper sides. Frame 2 also has a top resembling a thin rectangular plate, but its upper sides bear two small cylinders on their outer surfaces. These small rectangles and cylinders serve to facilitate heat transfer contact with the heating cylinder and to provide structural stability. The bottom of each frame contacts the cylinder via a rectangular profile. In Figure 5, (a) depicts the cruciform frame 1, (b) depicts the cruciform frame 2, and (c) displays the complete assembly of (a) and (b).

**Figure 6:**
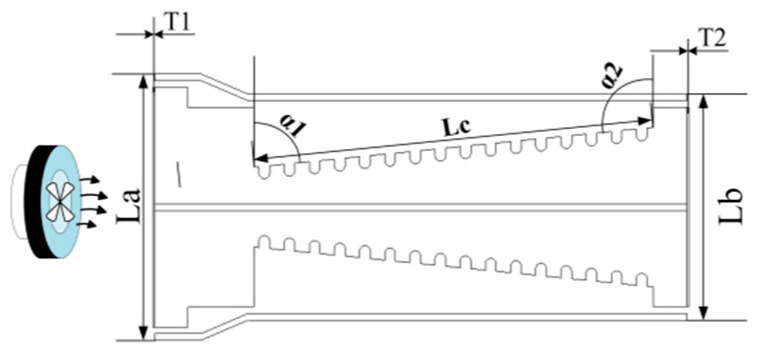
Flow channel structure and thermodynamic analysis model of the heating cylinder and cruciform frames.

According to prior research, the real-time quantitative PCR instruments with hot air circulation and temperature field regulation require exceptionally fast temperature responsiveness, in addition to temperature uniformity and stability. Hence, this study utilizes cruciform frames combined with a heating cylinder to establish the necessary temperature control system for the PCR instruments. As shown in Figure 6, this figure illustrates the flow channel structure and thermodynamic analysis model of the heating cylinder combined with the cruciform frames.

The figure depicts the core structure of the temperature control system for the real-time quantitative PCR instrument developed in this study, specifically the thermodynamic analysis model of the heating cylinder integrated with the cruciform frames (including the upper and lower sloped flow channels and their corresponding structural parameters). The definitions and design rationale for the key parameters in the model are as follows. The top lateral length (L_a_ = 36 mm), bottom lateral length (L_b_ = 30 mm), and top/bottom flow channel thicknesses (T_1_ = T_2_ = 5 mm) correspond to a flow channel design that is “wider at the top and narrower at the bottom”, which can optimize airflow velocity distribution based on the principle of fluid continuity. The slope length (L_c_ = 60.3 mm) ensures a balance between heat exchange sufficiency and airflow resistance. The angles between the upper slope and the horizontal plane (α1 ≈ 84.29°) and the lower slope and the horizontal plane (α2 ≈ 95.71°) are nearly 90° (asymmetrical). This design enhances the vertical impact of airflow on the reaction chamber while avoiding turbulent dead zones at the bottom. The synergistic design of the aforementioned parameters provides structural and fluid dynamic support for the temperature control system to achieve rapid heating/cooling rates and uniform temperature distribution.

Exploiting the principles of thermodynamics and fluid dynamics, while considering the rapid temperature cycling and uniformity requirements on nucleic acid amplification, the core parameter design and rationale for the cruciform frames are outlined as follows:

To achieve uniform heat exchange within the reaction chambers, a “wide top, narrow bottom” flow channel design was adopted, primarily based on the continuity equation for fluids and the formula for flow channel area:

(1) $$Q=A_{1}v_{1}=A_{2}v_{2}$$

(2) $$A_{t}=L_{a}\times T_{1}$$

(3) $$A_{b}=L_{b}\times T_{2}$$

where Q represents airflow volume, A denotes flow channel area, and v signifies airflow velocity; L_a_ = 36 mm (top transverse length), L_b_ = 30 mm (bottom transverse length), T_1_ = T_2_ = 5 mm (top/bottom thickness). Since L_a_ > L_b_, the bottom airflow velocity increases according to Equation (1). This design guarantees uniform heating wire distribution through the wider top channel, while enhancing convective heat transfer via the narrowed bottom airflow, thereby reducing heat loss.

Optimization was performed using the following friction loss formula:

(4) $$h_{f}=\lambda \frac{L_{c}}{D_{h}}\frac{v^{2}}{2g}$$

where λ signifies the friction factor, D_h_ represents the hydraulic diameter, g denotes the gravitational acceleration, and L_c_ is the slope length. It is essential for L_c_ to strike a balance between “adequate heat exchange” and “controllable resistance”. Setting L_c_ = 60.3 mm ensures sufficient space for heating wire arrangement and thermal radiation area, while also providing ample heat exchange time (t = L_c_/v) for the airflow in the ramp section. Consequently, the airflow undergoes thorough regulation before acting on the reaction chambers, thereby accelerating the temperature responsiveness.

To intensify the vertical impact of airflow within the reaction chambers, the following angles were designed using the velocity component formula:

(5) $$v_{y}=v\cos\alpha$$

(6) $$v_{x}=v\sin\alpha$$

α1 ≈ 84.29° (upper slope angle relative to the horizontal) and α2 ≈ 95.71° (lower slope angle relative to the horizontal). Both angles approximate 90°, thereby maximizing vy to reduce the lateral airflow dispersion. Meanwhile, a deviation of around 5° prevents turbulent dead zones at the bottom, achieving a balance between the airflow guidance efficiency and the flow field uniformity.

The transverse dimensions and L_c_ are critical for ensuring heat transfer efficiency, while the near-vertical angles enhance directed convection. The design of “wide-top, narrow-bottom” flow channel and angles enable uniform air distribution, thereby preventing localized temperature anomalies. L_c_ and angle parameters control D_h_ via Equation (4), balancing the air velocity with the fan power consumption. The asymmetry of α1 and α2, along with their protruding dimensions, adapt to the heating cylinder space, ensuring proper frame centering and uniform wire distribution. In summary, parameters have been comprehensively optimized based on the heat transfer efficiency, fluid characteristics, and amplification requirements, attaining the primary objectives of rapid temperature cycling and temperature uniformity through a well-designed system.

PCR requires not only rapid heating but also rapid cooling and uniform thermal distribution. The integration of fans and motors directly impacts the temperature control accuracy and reaction efficiency. The variable-speed feature of servo motor ideally suits the multi-temperature stage demands of PCR, providing low-speed airflow during heating and high-speed airflow during annealing. The uniform airflow distribution ensures consistent amplification efficiency across all reaction chambers, thus enhancing the experimental reproducibility.

**Figure 7:**
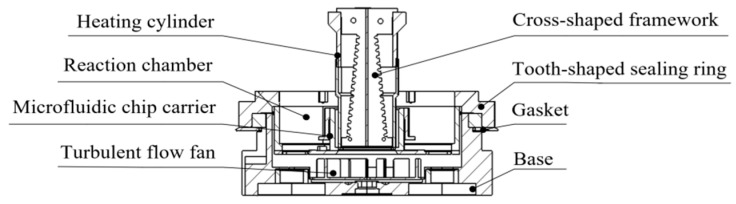
Complete temperature control system.

Accordingly, the entire temperature control system comprises a heating cylinder and a turbulator fan. The heating cylinder features a cruciform frame structure and heating wires within its copper cylindrical shell. The top of the cylinder is connected to an airflow rectification system, while the bottom is positioned vertically above the turbulator fan, creating an enclosed heating space for rapid temperature rise. The turbulator fan, installed beneath the microfluidic chip mounting slot, is directly connected to a servo motor. The servo motor controls the fan speed, enabling uniform heating and rapid cooling within the reaction chambers. Figure 7 illustrates the complete temperature control system for PCR instruments.

**Figure 8:**
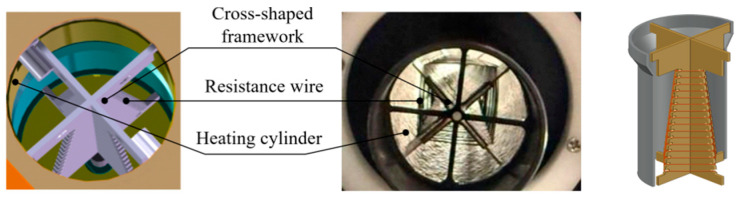
Heating wires.

Heating wires, made of Cr20Ni80 alloy, are wound in a square spiral pattern around the serrated grooves of the cruciform frames. They present a nested square spiral appearance when viewed from the top. The entire assembly is installed inside the cylindrical container [25,26]. Figure 8 displays the simulated structure and actual configuration of the cruciform frames with the heating wires wound around them.

When the temperature requirement increases, the wires become activated to raise the heating cylinder temperature. The air output from airflow rectification system passes through the heating cylinder to the top of turbulator fan. Controlled by a servo motor, the turbulator fan evenly distributes the heated air throughout the microfluidic chip reaction chamber, thereby ensuring that the chip is heated uniformly for rapid and thorough reactions.

### 3.2. Airflow Rectification System

**Figure 9:**
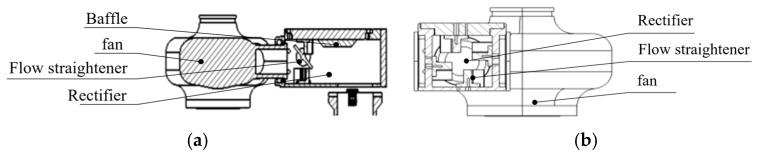
Airflow rectifier. (**a**) The complete rectification system. (**b**) Structure of four rectifier plates.

The airflow rectification system functions as the core auxiliary unit ensuring rapid temperature response in the PCR instruments, as well as uniform heating of the microfluidic chip. This study utilizes a baffle plate made of stainless steel wire mesh and rectifier plates made of polyether ether ketone. Figure 9a illustrates the complete airflow rectification system.

The rectifier plates undertake the primary rectification function, while the baffle plate handles secondary rectification. The structural design incorporates a trapezoidal baffle and four rectifier plates. The trapezoidal baffle guides the airflow into regular patterns and creates a gradient rectification effect, thereby effectively resolving the airflow stability issue during air blower speed fluctuations. The four rectifier plates, through an omnidirectional coverage design, thus converting disordered airflow into regular one. Combined with the guiding effect of the trapezoidal baffle, the airflow entering the heating cylinder becomes uniform in direction and stable in velocity, ultimately achieving the temperature difference control within ±0.1 °C across all regions of the microfluidic chip. Figure 9b provides a detailed structural schematic of the four rectifier plates.

Additionally, two hot-film anemometers are installed inside the rectification system, located at its air outlet to measure the airflow output, thereby facilitating precise temperature regulation.

## 4. Harmonic Response Analysis of the Temperature Control System

The reaction chamber of the real-time quantitative PCR instrument designed in this study exhibits excellent carrier compatibility, allowing flexible adaptation to various mainstream reaction carriers, such as microfluidic chips and 96-well plates. Great carrier compatibility provides a diverse range of options for selecting carriers in performance analysis. Considering experimental requirements and technological validation objectives, microfluidic chip is selected as the primary subject for discussing core performance analysis. The underlying rationale lies in the high alignment between microfluidic chips and the practical application needs of PCR detection as well as the technical characteristics of the temperature control system.

This study selects the real-time quantitative PCR instrument developed by Tianlong Technology, a leading enterprise in genetic testing and molecular diagnostics, as the benchmark for comparison. This stems from its exemplary status within the industry, its comprehensively advanced technical performance, and its ability to flexibly adapt to diverse scenarios. Such full-scenario adaptability is attributed to miniaturization and high-integration designs enabled by micro-mechanical technology, along with the miniaturization of core components within the temperature control module, optical module, and control module. While ensuring performance, Tianlong’s instruments achieve optimized size, with portable models facilitating flexible deployment in on-site testing scenarios. Its technical solutions and performance parameters have become a widely recognized reference standard in the industry. Tianlong’s instrument is selected as the comparison object, ensuring the scientific rigor, reference value, and industrial significance of the evaluation. Firstly, Tianlong’s technological approach and performance metrics represent the current mainstream high standards of commercial PCR instruments, aligning closely with the core performance evaluation dimensions of the instrument presented in this study. Direct comparison of data objectively reflects the competitiveness of this instrument in key indicators. Secondly, there is a substantial overlap between the application scenarios covered by Tianlong’s instruments and the design positioning of this study’s instrument, clearly highlighting the differentiated advantages of this instrument under the hot air circulation temperature control technology and micro-mechanical integration scheme. Thirdly, as a mature product validated by tens of thousands of institutions, Tianlong’s instruments provide reliable reference benchmarks through their publicly available performance parameters, certifications, and market feedback, thereby avoiding the issue of insufficient persuasiveness due to a lack of industry recognition of the comparison object. Finally, this benchmark brand not only directly verifies the technological advancement and engineering feasibility of this study’s instrument, but also demonstrates the iterative upgrades in core PCR technologies through data comparison, offering valuable insights for the industry’s technological development.

From the perspective of experimental resources and cost, the upgraded reaction chamber utilizing microfluidic chips can reduce sample and reagent consumption to one-tenth or even less compared to traditional 96-well plates. Employing microfluidic chips is particularly crucial for detecting clinically scarce samples, as it prevents the waste of precious samples while significantly lowering reagent costs. This approach better aligns with the practical demands of low consumption and high efficiency in precision detection scenarios. Regarding the performance adaptability of the temperature control system, the micro-scale structure of microfluidic chips significantly shortens the heat transfer path, enabling faster thermal response times for the reaction system. This precisely matches the rapid thermal cycling characteristics of this temperature control system, which achieves heating and cooling rates of 7.5 ± 0.1 °C/s and 13.5 ± 0.1 °C/s, respectively. If larger traditional carriers were used, heat transfer delays would mask the rate advantages of the temperature control system, making it difficult to accurately verify the actual efficacy of the hot air circulation and temperature field regulation technologies. Regarding experimental result stability and reliability, the enclosed microchannel network and independent reaction chamber design of microfluidic chips allow precise control of the reaction microenvironment. On the one hand, fluid and temperature distribution across channels can remain highly uniform under the regulation of the temperature control system, avoiding the temperature gradient discrepancies between edge and center wells common in traditional carriers. On the other hand, the closed system and independent chambers effectively prevent cross-contamination between samples and reduce the risk of aerosol contamination. For high-sensitivity detection methods like fluorescent quantitative PCR, this significantly minimizes interference from false-positive results, ensuring the authenticity of performance analysis data. Furthermore, the anti-interference capability of microfluidic chips more accurately reflects the actual performance of core indicators such as temperature field uniformity and thermal cycling stability of the temperature control system, avoiding the influence of non-specific factors on experimental conclusions. For performance comparison validation, this study selects the 96-well plate as a reference carrier, adopting the industry-standard practice in PCR detection. The 96-well plate possesses a broad baseline of experimental data and methodological acceptance. Benchmarking against the performance of 96-well plate-compatible equipment allows rapid validation of the detection accuracy of this temperature control system paired with the microfluidic chip solution, while providing an intuitive assessment of its repeatability. This approach offers quantifiable reference basis for selecting application scenarios involving different carriers.

### 4.1. Thermal Performance of PCR Instruments

A comparative investigation was conducted around the thermal performance of a real-time quantitative PCR instrument that employs hot air circulation and temperature field regulation. Initially, performance benchmarking was performed against two mainstream counterparts: conventional liquid metal bath and Peltier cooler systems. Under identical experimental conditions, experimental data were collected using type T thermocouple probes and systematically analyzed through simulation techniques.

The results demonstrate that the designed PCR instrument, which incorporates hot air circulation and temperature field regulation, exhibits considerable advantages in key thermal performance metrics: The up ramp reaches 7.5 ± 0.1 °C/s, whereas the down ramp attains 13.5 ± 0.1 °C/s, representing respective improvements of 6.25 times and 16.9 times over the liquid metal bath amplifier, and 5 times and 12.3 times over the Peltier-cooled air bath amplifier. The steady-state temperature fluctuation is limited to ± 0.1 °C, surpassing the liquid metal bath (±0.3 °C) and Peltier cooler (±0.4 °C) counterparts. A single PCR cycle takes 28 ± 2 s, with 35 cycles completed in 16.3 ± 0.6 min, indicating a 1.9-fold reduction in total cycle duration compared to the liquid metal bath amplifier. This benefit primarily arises from the synergistic effect of the airflow rectification system and the heating cylinder temperature control structure. The significantly improved heat exchange efficiency fully satisfies the core requirements for temperature control efficiency and stability in rapid nucleic acid testing. Detailed comparative results of the key thermal performance metrics are presented in Table 1.

**Figure 10:**
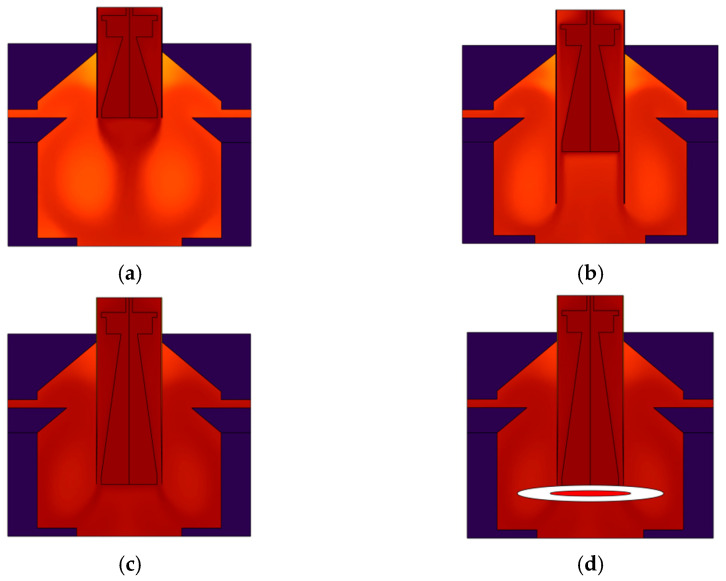
Simulation diagrams of different heating cylinder configurations. (**a**) Equal height short cylinder configuration. (**b**) Unequal height cylinder configuration. (**c**) Equal height long cylinder configuration. (**d**) Microfluidic chip position within reaction chamber.

Meanwhile, to examine the influence of structural design on thermal performance, this study designs three heating chamber configurations: a short cylinder with the cruciform frames and heating chamber at equal heights, a long cylinder with the cruciform frames and heating chamber at equal heights, and a configuration featuring cruciform frames and heating chamber at unequal heights. These were compared under consistent conditions of 80 W heating power and 2000 r/min fan speed. Figure 10 displays the simulation diagrams for various configurations: (a) short cylinder with equal height, (b) unequal height configuration, (c) long cylinder with equal height, and (d) microfluidic chip position within reaction chamber, which showcases uniform heating throughout the chip.

The experimental data reveal that the long cylinder structure, which features cruciform frames and heating chamber at equal height (Scheme C), exhibits optimal overall thermal performance. Specifically, the temperature uniformity deviation within reaction chambers is only 0.1 °C, which is about one-third that of the rectangular structure (Scheme B); the response time for heating to 95 °C is 14 ± 1 s, while cooling down to 55 °C takes 9 ± 1 s, representing respective reductions of 23.6% and 14.3% compared to the Schemes B and A; after 35 PCR cycles, the sample amplification efficiency reaches 98.9 ± 0.2%, which significantly surpasses that of Scheme A (92.5 ± 1.3%) and Scheme B (88.3 ± 1.5%). The core mechanism behind Scheme C’s performance advantage lies in the synergistic interaction between the cruciform frames and the equal height long cylinder structure. This design effectively guides hot air into the reaction chambers, where it forms an ordered flow field by the action of turbulator fan. The ordered airflow evenly permeates the surface of the microfluidic chip, thus efficiently suppressing the formation of dead air zones in the chamber corners, as well as axial temperature gradients. Additionally, this structural configuration reduces the dead volume within the chambers, and lowers the system thermal load, ultimately achieving a marked improvement in nucleic acid amplification efficiency through the optimization of temperature uniformity and ramp rates. Thermal performance comparisons across different structural configurations are described in Table 2.

**Figure 11:**
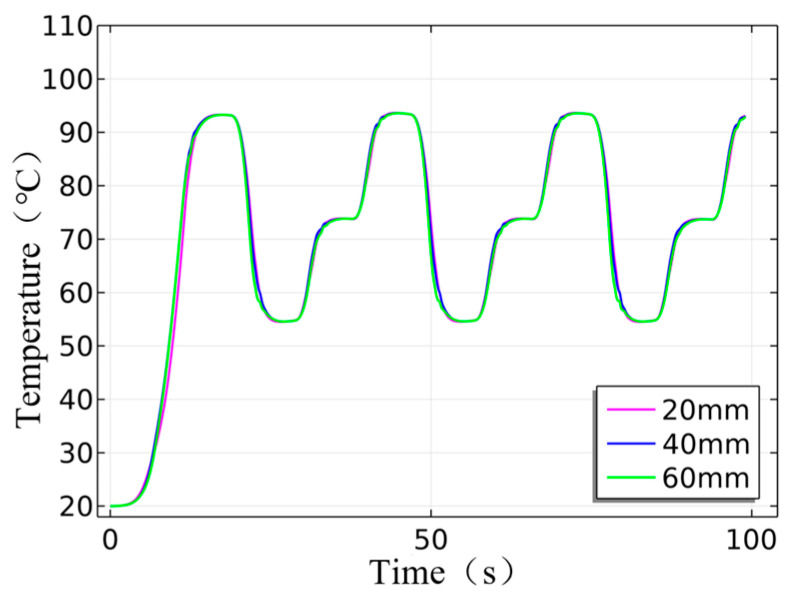
Temperature curves at different heights of the cruciform frames.

Figure 11 displays the temperature curves for the cruciform frames at different heights, with the pink curve representing the temperature curve at 20 mm of the heating cylinder, the blue curve at 40 mm, the green curve at 60 mm, and comparing the temperatures at various heights. Clearly, the temperature uniformity inside the heating cylinder is high, and uniform heating is maintained during circulation without fluctuations.

**Figure 12:**
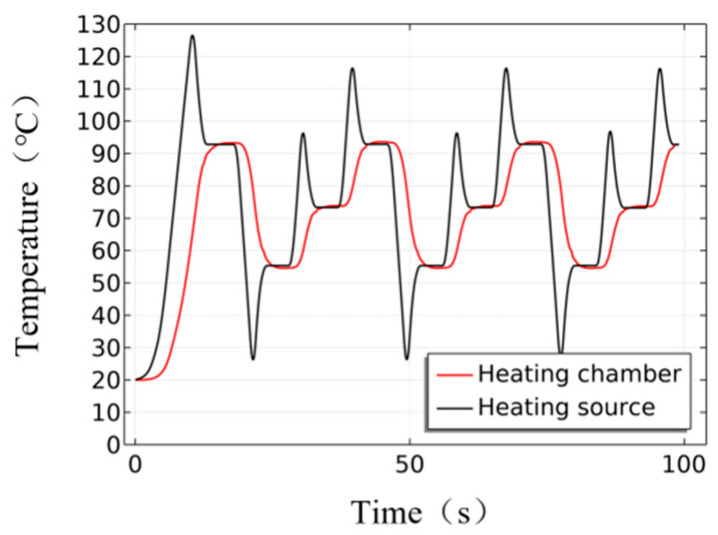
Temperature comparison between heating chamber and heating source.

The temperatures of the heating chamber versus heating source of the heating cylinder are illustrated in Figure 12. The heating source exhibits rapid response, while the overall temperature variation within the heating chamber is kept uniform. The speed and stability of temperature cycling remain consistent even after multiple cycles.

**Figure 13:**
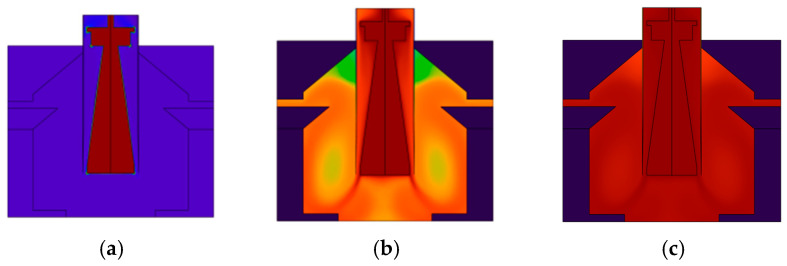
Reaction chamber temperatures at different time points. (**a**) 0 s. (**b**) 15 s. (**c**) 29 s.

The heating process of a real-time quantitative PCR instrument incorporating hot air circulation and temperature field regulation is depicted in Figure 13, with (a) indicating the temperature at the start of heating; and (b) and (c), respectively, indicating the chamber temperatures after 15 s and 29 s of heating.

The proposed PCR temperature control system was compared with the Gentier 96R PCR system (Tianlong) manufactured by Xi’an Tianlong Technology Co., Ltd. in Xi’an City, Shaanxi Province, China in terms of ramp rates, temperature uniformity, and temperature stability.

**Figure 14:**
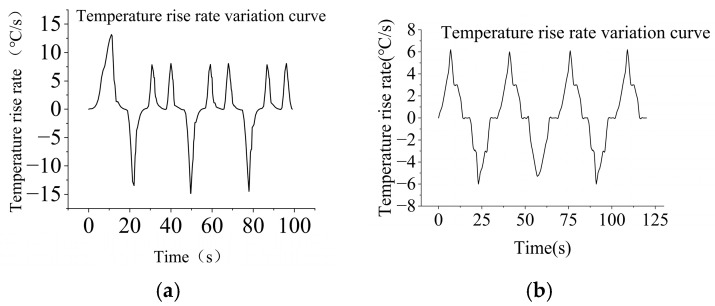
Comparison of ramp rate curves. (**a**) The proposed PCR instrument. (**b**) Gentier 96R PCR system.

After multiple experimental tests under specific conditions, the proposed PCR instrument achieves an up ramp of 7.5 °C/s, while the current Gentier 96R PCR system has an up ramp of 5.5 °C/s. As shown in Figure 14a, the up ramp of the proposed PCR instrument reaches up to 13.1 °C/s during the initial heating phase. Upon attaining the target temperature, the average up ramp is as high as 7.5 °C/s, whereas the average down ramp is 13.5 °C/s. Both up and down ramps remain consistently stable, completing one full cycle in 28 s. The 0 °C/s up ramp between two peaks signifies the isothermal holding time required during the heating process. Figure 14b illustrates the ramp rate data for the Gentier 96R PCR system. The initial heating phase reveals a peak up ramp of 6.2 °C/s, and after reaching the target temperature, the average up ramp is as high as 6.1 °C/s, whereas the average down ramp is 5.5 °C/s, with one full cycle completed in 38 s.

Temperature uniformity, as a key indicator for evaluating the core performance of real-time quantitative PCR instruments, directly impacts the consistency of nucleic acid amplification reactions and the accuracy of detection results. The temperature control performance of microfluidic chip was tested, revealing that the designed instrument consistently maintains a temperature deviation within ±0.1 °C, matching the ±0.1 °C deviation of the Gentier 96R PCR system. This small temperature deviation ensures that the amplification reactions take place under uniform temperature conditions across all microfluidic chip chambers. Consequently, the experimental errors caused by temperature variations are effectively mitigated, thus greatly enhancing the reliability and reproducibility of detection results.

**Figure 15:**
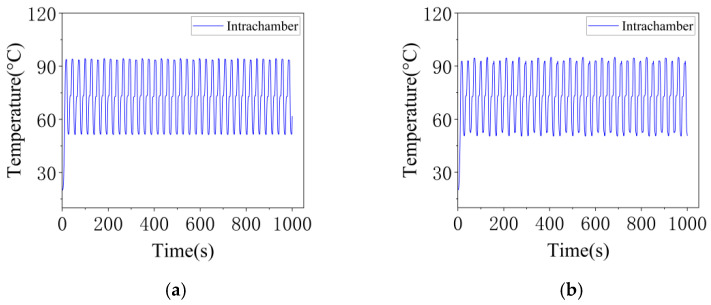
Stability comparison during multiple cycles. (**a**) The designed PCR instrument. (**b**) Gentier 96R PCR system.

In prolonged, continuous nucleic acid amplification experiments, temperature stability is a critical factor guaranteeing reliable detection results. The PCR instrument, designed with hot air circulation and temperature field regulation, incorporates a high-precision temperature feedback control system. This system enables real-time monitoring and dynamic adjustment of chamber temperature, thereby effectively suppressing fluctuations. The experimental data confirms that during continuous operation for several hours, the designed instrument stably maintains temperature fluctuations within ±0.1 °C. The Gentier 96R PCR system also exhibits temperature fluctuations within ±0.1 °C. Figure 15a presents the temperature stability curve inside the chambers during multiple PCR cycles for the designed instrument; (b) displays the corresponding temperature variation curves inside and outside the chambers for the Gentier 96R PCR system.

### 4.2. Rectification Performance of PCR Instruments

To systematically evaluate the airflow impact on the heating efficiency, experiments were conducted using typical nucleic acid samples. The procedures strictly adhered to standardized protocols. Initially, the nucleic acid samples were evenly placed within the reaction module of the proposed PCR instrument, with initial and target temperature parameters set. Under static condition, the internal air blower, rectifier, and turbulator fan were deactivated to create a relatively static thermal environment. Following the initiation of heating program, sample temperatures were recorded at fixed intervals. Thereafter, the above experimental steps were repeated under airflow conditions. All other variables were strictly controlled throughout the experimentation to ensure the scientific validity and reproducibility of results, thus providing a reliable basis for subsequent data analysis and conclusion derivation.

**Figure 16:**
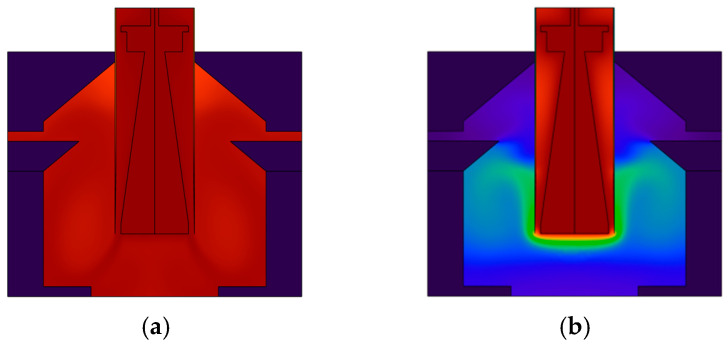
Up ramp curves. (**a**) Up ramp under airflow condition. (**b**) Up ramp under static condition.

**Figure 17:**
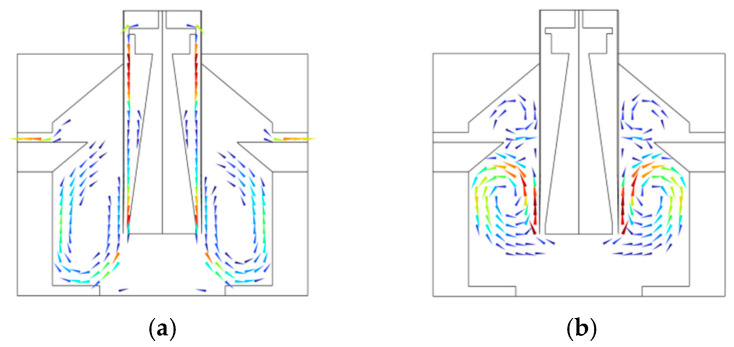
Fluid flow patterns. (**a**) Fluid flow under airflow condition. (**b**) Fluid flow under static condition.

Up ramp characteristics: The experimental data indicates that under the airflow conditions, the up ramp is significantly enhanced for the proposed PCR instrument. The time required to reach the target temperature from the initial temperature is only 0.5 min under the airflow conditions, compared to 1.5 min under the static conditions. The fan operation accelerates the air circulation, facilitating the rapid transfer of hot air to the sample surface, which enhances the convective heat transfer effect, and thereby expedites the sample heating process. Relevant characteristic curves alongside flow field distributions are depicted in Figure 16 and Figure 17. Figure 16a presents the up ramp curve under airflow conditions, whereas b displays that under static conditions. Figure 17a and b, respectively, illustrate the flow field distributions under airflow and static conditions.

In Figure 17a, the arrows indicate the direction of airflow movement. The colors transition gradually from dark blue through blue, cyan, green, yellow, and orange to deep red, where cool-toned regions correspond to low-velocity airflow and warm-toned regions correspond to high-velocity airflow, clearly illustrating the distribution of high-velocity airflow in the central channel and the velocity variation pattern in the recirculation zones on both sides. In Figure 17b, due to the extremely low airflow velocity, the transition from cool to warm tones corresponds to a slight increase in airflow velocity from low to high, fully demonstrating the velocity gradient and distribution characteristics of the swirling airflow on both sides of the reaction chamber.

According to the monitoring results of multi-point temperatures within the reaction module, the temperature distribution under airflow condition is considerably more uniform than that under static condition. In the static environment, the sluggish airflow leads to heat transfer dominated by conduction, which results in noticeable temperature gradients across different regions of the reaction module, with deviations reaching ±0.5 °C. In the airflow environment, forced convection enables uniform heat diffusion, controlling the temperature deviations within ±0.1 °C, a range that is significantly narrower than the ±0.5 °C observed in the static setting. A uniform temperature field ensures that all samples undergo amplification under consistent thermal conditions, thus effectively reducing the experimental bias introduced by temperature variations to enhance the consistency and reliability of results.

**Figure 18:**
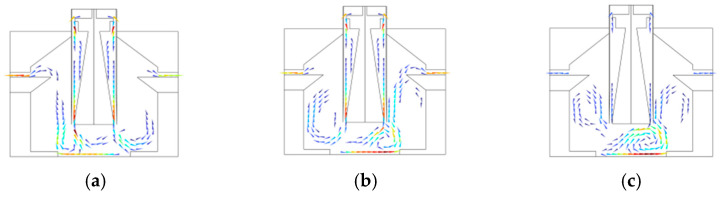
System operation results under airflow without rectification. (**a**) Fluid flow pattern at 0.1 m/s. (**b**) Fluid flow pattern at 0.2 m/s. (**c**) Fluid flow pattern at 0.3 m/s.

To validate the effectiveness of the rectifier structure, comparative experiments with and without rectification were conducted, and the results are illustrated in Figure 18. Figure 18a, b, and c, respectively, depict the flow field distributions at disordered airflow velocities of 0.1 m/s, 0.2 m/s, and 0.3 m/s. It is evident that the direct output of disordered airflow from the air blower interacts with the turbulator fan at the bottom of reaction chambers, readily creating stagnant air pockets and flow turbulence, which ultimately leads to an uneven distribution of chamber temperatures.

In Figure 18, the arrows indicate the direction of airflow movement. The colors transition gradually from dark blue through blue, cyan, green, yellow, and orange to deep red, where cool-toned regions correspond to low-velocity airflow and warm-toned regions correspond to high-velocity airflow, clearly illustrating the airflow distribution and the variation pattern of airflow velocity in the reaction chamber.

**Figure 19:**
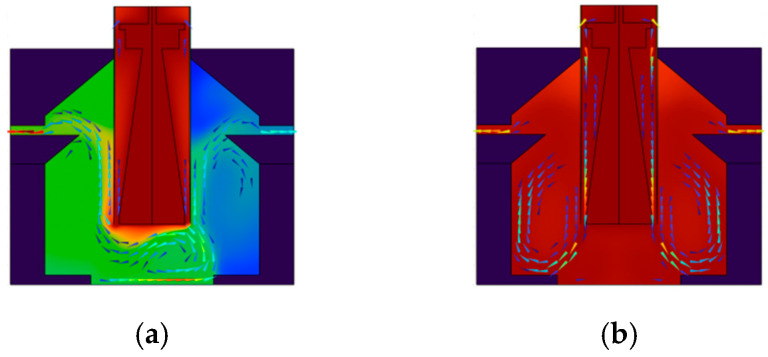
Temperature fields with and without airflow rectification. (**a**) Temperature fields without airflow rectification at 30 s. (**b**) Temperature fields with airflow rectification at 30 s.

During the operation of real-time quantitative PCR instruments employing hot air circulation and temperature field regulation, the temperature uniformity within the reaction chambers is a critical determinant for the amplification specificity and result reproducibility, while the introduction of a rectification system significantly improves this metric. For a PCR device lacking a rectification system, the temperature field evolution within its reaction chambers exhibits pronounced imbalances. As shown in Figure 19a depicting the temperature field distributions from 30 s, dead zones for heat transfer are formed around the chamber periphery due to the presence of stagnant air pockets. Chaotic boundaries between high- and low-temperature zones are already noticeable. The low-temperature state in the air pocket zones persists without relief. The chambers still fail to establish a uniform temperature field. This spatial temperature heterogeneity directly contributes to variations in amplification rates and enzyme activity across different regions within the reaction system, ultimately compromising the reliability of the detection results.

Following the implementation of the rectification system, the temperature field characteristics of the reaction chambers undergo fundamental improvement. Figure 19b display the temperature field diagrams at 30 s. It is evident that the rectification system effectively eliminates the air pockets by regulating the airflow paths: the airflow follows an orderly trajectory along the chamber sidewalls, with temperature gradients exhibiting a regular distribution; the temperature differences around the chamber periphery are significantly reduced, with no discernible low-temperature stagnation zones; a uniform and stable temperature field is formed throughout the reaction chambers and their surrounding areas, thereby achieving synchronized temperature changes across all regions. Consequently, both spatial consistency and temporal stability of the thermal field are markedly improved.

The rectification system’s mechanism for regulating the temperature field essentially works by optimizing the airflow direction, thereby eliminating the stagnant air pockets present in the non-rectified state. This allows hot air to fully cover all chamber regions, enhancing both the spatial uniformity and temporal synchrony of heat exchange. Consequently, time differences in reaching target temperatures across chamber regions are reduced, while also guaranteeing that the nucleic acid amplification reactions occur in a homogeneous thermal environment.

By comparing the PCR performance between the designed temperature control system and the Gentier 96R PCR system, and further investigating the heating effects of the designed system under conditions with or without airflow and with or without airflow rectification, several noteworthy research findings were obtained. The comparisons demonstrate the advantages of the designed device in key performance metrics such as up ramp, temperature uniformity, and temperature stability, particularly highlighting its competitive edge regarding the PCR amplification efficiency under high-temperature conditions. This confirms that the proposed temperature control system represents a significant technological advancement, offering efficient and precise temperature control for nucleic acid amplification experiments. The comparisons of heating effects with and without airflow in the device, and with and without airflow rectification, clearly demonstrates the crucial role of fan operation in enhancing the up ramp and temperature uniformity, which provides vital technical support for the optimized design and practical application of PCR devices.

## 5. Reliability Testing of the Temperature Control System

For the comparative experiments, a real-time fluorescent quantitative PCR instrument from Tianlong Technology was selected as the benchmark. The two systems share a high degree of consistency in the core PCR workflow, ensuring a scientifically valid comparison. Regarding the reaction principle and process, they strictly adhere to the standard PCR cycle logic of denaturation, annealing, and extension. The fundamental reaction mechanism is based on DNA semi-conservative replication. The entire amplification process is identical: denaturation at 94–95 °C to unwind double-stranded DNA, annealing at 55–65 °C for primer-template binding, and extension at 72 °C for new strand synthesis. In terms of working principle and process, both of them rely on a temperature control system to regulate the thermal cycling as the core framework, and their operational workflows include the standard steps of setting amplification protocols, loading reaction carriers, running thermal cycles, collecting temperature and fluorescence data, and analyzing amplification results. The experimental operation logic and data acquisition dimensions are also fully aligned.

Regarding performance evaluation and experimental validation, the definitions and verification methodologies for core performance indicators remain consistent, such as ramp rate, temperature control accuracy, amplification efficiency, and repeatability. Furthermore, the methods for assessing heating capability are identical. The only distinction lies in the core heating method of the temperature control system. The Tianlong instrument employs mature semiconductor-based temperature control technology, while the system presented in this study utilizes a hot air circulation and temperature field regulation approach to achieve thermal cycling. The two systems are highly consistent in the core logic of PCRs, workflow, and performance evaluation dimensions, differing only in the heating method. Therefore, selecting the Tianlong instrument minimizes interference from other variables, enabling a precise validation of the performance of the hot air circulation temperature control technology compared to conventional semiconductor-based control.

The system presented in this study can utilize either a microfluidic chip or a 96-well plate as the reaction carrier. There is no fundamental difference in the PCR experiment itself; the carriers merely adapt to different heating methods. However, the microfluidic chip offers advantages such as lower consumable consumption and stronger resistance to contamination and cross-interference. As a more advanced device, it better aligns with technological development trends. Consequently, microfluidic chips were ultimately selected as the core reaction carrier for these experiments. The Tianlong instrument, as an industry benchmark in the PCR field, has established its technical solutions and performance parameters as widely recognized reference standards. By comparing against it under conditions of high core consistency, this study not only enhances the authority of the system performance validation, but also provides more industry-relevant evidence for the application of hot air circulation temperature control technology in the PCR domain.

### 5.1. Experimental Setup

**Figure 20:**
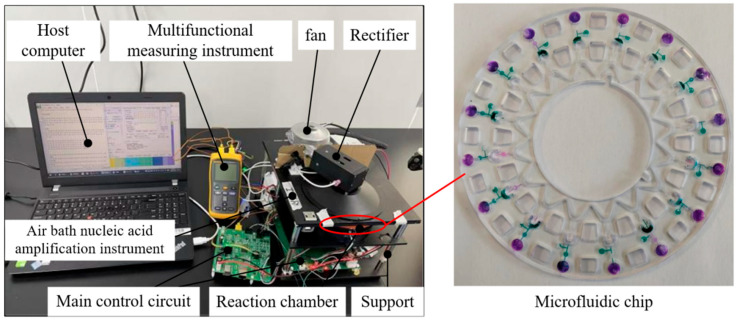
System test fixture.

Firstly, a system test fixture was constructed for the real-time quantitative PCR instrument with hot air circulation and temperature field regulation. The fixture comprises a host computer, a multifunctional measuring instrument, the real-time quantitative PCR instrument employing hot air circulation and temperature field regulation, and a main control circuit board. The main control circuit board handles data processing, program execution, and experimental monitoring; it is responsible for receiving program instructions, driving the heating module for temperature ramping, controlling temperature sensors for real-time feedback of temperature data, and coordinating the stable operation of all components. The host computer connects to the main unit of the real-time quantitative PCR instrument with hot air circulation and temperature field regulation. It is used to set amplification programs via specialized software, with the screen displaying the experimental program interface, data charts, and final amplified data analysis. Inside the reaction chamber, a microfluidic chip, after completing sample and reagent transfer and mixing operations, is loaded. The miniaturized microfluidic chip incorporates a micron-scale channel network, enabling precise and low-loss transport of nucleic acid samples and amplification reagents. The microfluidic chip precisely aligns with the positioning structure of the reaction chamber, and its ultra-thin thermally conductive substrate efficiently receives the thermal flow from the hot air circulation temperature control system, ensuring rapid thermal coupling and synchronous response between the reaction system and the temperature control module. In coordination with the specialized software of the host computer and the parameter monitoring of the multifunctional measuring instrument, synchronous acquisition, recording, and visual presentation of temperature control data and amplification fluorescence signals are achieved. Figure 20 illustrates the system test fixture.

### 5.2. Reproducibility Analysis

**Figure 21:**
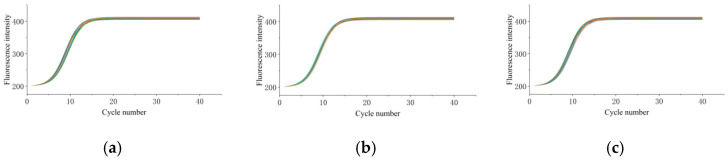
Repetitive amplification curves. (**a**) Chamber C2. (**b**) Chamber C7. (**c**) Chamber C15.

To systematically evaluate the reproducibility and operational stability of the designed PCR instrument employing hot air circulation and temperature field regulation, a series of experiments was conducted. Three chambers, C2, C15, and C7, were randomly selected on the integrated disk-shaped microfluidic chip. Each chamber received an equal volume of identical culture medium, followed by 35 independent amplification experiments, each involving 40 temperature cycles. The instrument performance was analyzed by monitoring the dynamic changes in fluorescence intensity. Figure 21a, b, and c, respectively, illustrate the amplification curves from the 35 repeated experiments for chambers C2, C7, and C15. Each curve in a different color represents the amplification curve of one amplification experiment. Clearly, the amplification curves across different chambers and multiple repetitions all exhibit highly overlapping, typical S-shaped growth trends, with remarkable consistency in key metrics such as peak time, fluorescence growth rate, and plateau fluorescence intensity. This suggests excellent reproducibility of the instrument across chambers and batches, with commendable chamber uniformity and batch reproducibility. It effectively mitigates the amplification biases caused by chamber differences and repeated operations, thereby providing reliable performance support for high-throughput, multi-batch experiments of disk-shaped microfluidic chips.

A further analysis of the amplification curve smoothness and plateau characteristics reveals exceptionally smooth transitions across all three sets of curves, without significant fluctuations or abnormal peak shapes observed. Moreover, the fluorescence intensity during the plateau phase remains at high and stable levels. This demonstrates that the instrument maintains precise and stable temperature control throughout 40 thermal cycles, consistently delivering a uniform environment for nucleic acid amplification. Consequently, the enzymatic reaction activity and template amplification stability are guaranteed, thus effectively suppressing the non-specific amplification and amplification efficiency fluctuations caused by temperature variations, ultimately ensuring the reliability of experimental results.

### 5.3. Temperature Uniformity Analysis

Temperature control performance is a core metric for achieving precise nucleic acid amplification in real-time quantitative PCR instruments. The linear correlation between set and measured temperatures, along with the spatial temperature uniformity within reaction chambers, directly determine the amplification efficiency, specificity, and result reproducibility. Based on the fitted temperature curves derived from actual instrument and simulation model, a systematic analysis of reaction chamber temperature characteristics was conducted.

**Figure 22:**
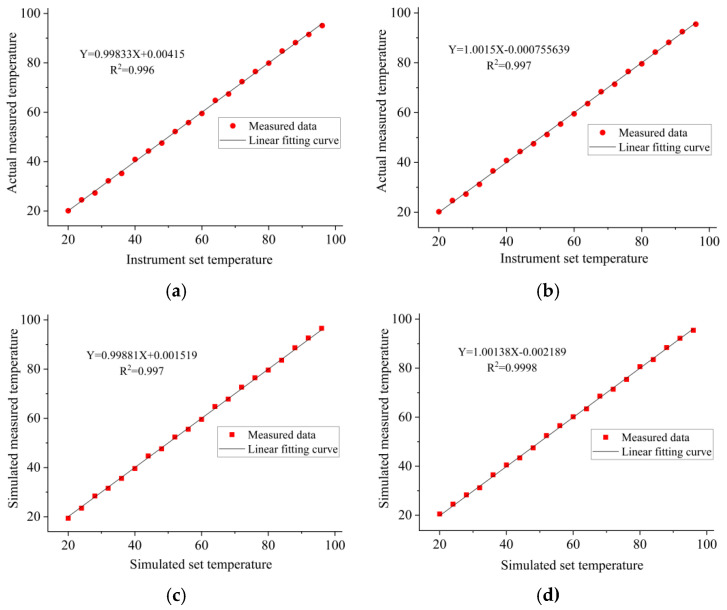
Linearly fitted temperature curves. (**a**) Linearly fitted curves for actual hybridization chamber. (**b**) Linearly fitted curves for actual reaction chamber. (**c**) Linearly fitted curves for simulated hybridization chamber. (**d**) Linearly fitted curves for simulated reaction chamber.

Figure 22a illustrates the temperature data within the nucleic acid hybridization chamber of an actual real-time quantitative PCR instrument, whereas Figure 22b shows the temperature data from the nucleic acid reaction chamber of the same instrument. Figure 22c presents the temperature data within the nucleic acid hybridization chamber of a simulated real-time quantitative PCR instrument, while Figure 22d displays the temperature data from the nucleic acid reaction chamber of the same simulated instrument. The PCR instruments exhibit exceptional linear correlation between the set and measured temperatures. According to the coefficients of determination (R^2^) derived from the four sets of fitted curves, the R2 values corresponding to the hybridization and reaction chambers in the actual device and simulation model all reach 0.99. This finding suggests a nearly perfect linear correspondence between the set temperature and actual measured temperature, with the linear fit of the temperature response approaching an ideal state.

The parameters of fitting equations were specifically analyzed. In the actual device, the fitting equations for the hybridization and reaction chambers are, respectively:

(7) Y = 0.99833X + 0.00415

(8) Y=1.0015X−0.000755639

In the simulation model, the respective fitting equations for the hybridization and reaction chambers are:

(9) Y = 0.99881X+0.001519

(10) Y=1.00138X−0.002189

The slopes of all curves approach 1, indicating a highly precise proportional relationship between the measured and set temperatures. Meanwhile, the absolute values of the intercepts remain within the 0.01 magnitude range, reflecting minimal temperature deviation within the system. This feature ensures stable output of actual temperatures that closely match the setpoints across different temperature ranges, thus providing fundamental assurance for the precise temperature control in nucleic acid amplification reactions.

The spatial temperature uniformity within the reaction chamber is outstanding. The analyzed chambers are located on the same horizontal plane of the microfluidic chip. The hybridization chamber is positioned between 31.5 and 35.2 mm from the chip’s radius, while the reaction chamber is located at 42 mm of the radius. Theoretically, this spatial separation between the two chambers could lead to an inhomogeneous temperature distribution. Nevertheless, comparison of the fitted parameters reveals that for the actual device, the slope difference between the two chambers’ fitted curves is only 0.0032, and the absolute difference in intercepts is approximately 0.01. For the simulation model, the slope difference between the two chambers further narrows to 0.0026, and the absolute difference in intercepts is less than 0.003. This finding confirms that the temperature response characteristics at different radial positions within the same horizontal plane are remarkably consistent, indicating a high degree of uniformity in the internal spatial temperature distribution. For nucleic acid amplification reactions, this uniformity effectively prevents fluctuations in amplification efficiency arising from differing sample positions within the chambers, thereby ensuring the consistency and reliability of multi-sample detection results.

### 5.4. Reliability Analysis

**Figure 23:**
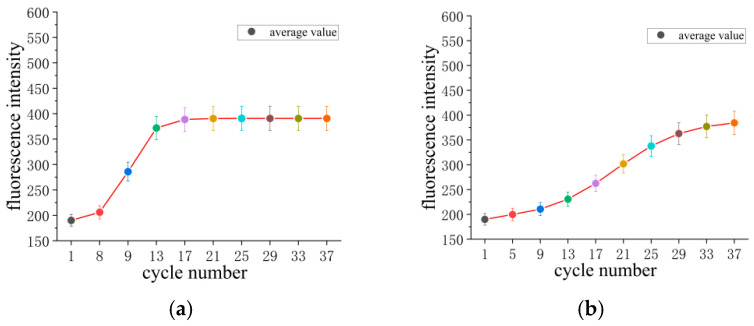
Fluorescence intensity boxplots. (**a**) The proposed PCR Instrument. (**b**) Gentier 96R PCR system.

According to Figure 23, the PCR instrument employing hot air circulation and temperature field regulation demonstrates significant advantages in reliability. After ≥5 cycles, its median fluorescence intensity and distribution range surpass those of the Gentier 96R PCR system. The median fluorescence intensity at cycle 13 reaches approximately 380 RFU for the proposed instrument, whereas is only about 220 RFU for the Gentier 96R PCR system, indicating a substantial enhancement in fluorescence intensity. This signifies that the temperature within the reaction chamber satisfies the nucleic acid amplification requirements. The proposed instrument demonstrates an accelerated increase in fluorescence intensity with the cycle number, maintaining a stable, high fluorescence plateau in the later cycles without notable signal decay. A final comparison with the Gentier 96R PCR system reveals consistency in fluorescence intensity.

In Figure 23, the red curve visually illustrates the overall trend of fluorescence intensity increasing with the number of cycles. The dots of different colors correspond to the average real-time fluorescence intensity at each cycle, where the transition from cool to warm tones also reflects the change in fluorescence signal from weak to strong. This clearly demonstrates the growth pattern of fluorescence signals and the characteristic of intensity stability during the plateau phase in PCR reactions.

**Figure 24:**
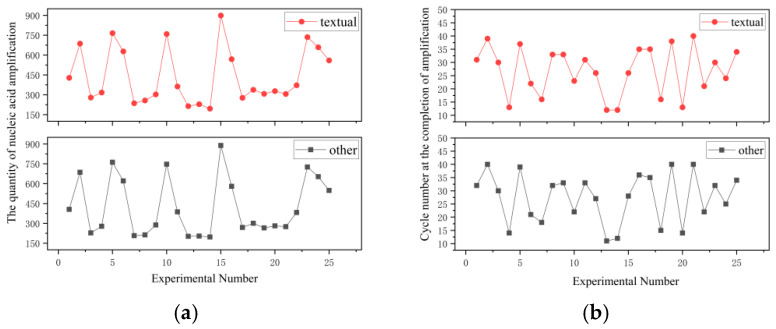
Distribution of amplification correlation data. (**a**) Amplification capability correlation. (**b**) Amplification time correlation.

Select 22 groups of nucleic acid samples with different copy numbers, and precisely load each group of samples into the corresponding sites of the reaction chambers in the microfluidic chip and then subjected to amplification within a heating chamber. The proposed PCR instrument and Gentier 96R PCR system were used to separately conduct 25 amplification tests, and the correlation between the two methods was examined, yielding the test data curves shown in Figure 24. The analysis reveals that the designed PCR instrument has a correlation greater than 0.997 with the Gentier 96R PCR system, which fulfills the design specifications.

## 6. Conclusions

Research on a temperature control system for real-time quantitative PCR instruments, which is based on hot air circulation and temperature field regulation, has been presented. The study focuses on a heating cylinder to function as heating elements for the PCR instruments, which remarkably enhances the stability and responsiveness of temperature control performance, ultimately guaranteeing that the PCR instruments achieve both rapid temperature cycling and reaction chamber temperature uniformity. Through performance testing of the heating structure, the ramp rates and temperature stability within the heating chamber during multiple cycles are analyzed, finding that the system achieves a temperature stability of ±0.1 °C and a rapid response time of ±0.05 s. The test results for the rectifier assembly and heat transfer/dissipation system indicate an up ramp of 7.5 °C/s, a down ramp of 13.5 °C/s, and temperature uniformity of ±0.1 °C. All metrics meet the design requirements established in this study and comply with the national standards for real-time quantitative PCR instruments. Conclusively, the accuracy of the designed system fully aligns with the operational requirements of real-time quantitative PCR instruments. Measurement data also corroborates that the developed hot air circulation and temperature field regulation system achieves its design objectives regarding rapid temperature cycling, uniformity within reaction chambers, temperature control stability, and rapid responsiveness. In addition to providing core technological support for improving the development processes of such PCR instruments, the proposed temperature control method is also applicable to the temperature or uniformity control and calibration tasks in other precision instruments. The temperature control system effectively addresses the bottlenecks of insufficient temperature control efficiency and large size in traditional PCR instruments, providing a high-performance technical solution for diversified scenarios such as point-of-care testing. The achievement of these results relies on the miniaturized design, precise integration, and rapid response characteristics of the core functional components: the microfluidic chip’s miniature reaction unit, the precise channel structure of the cruciform miniature skeleton, the collaborative drive of the miniature turbofan and servo motor, and the real-time monitoring of the PT100 miniature temperature sensor and thermal film anemometer. It is precisely by leveraging the advantages of micro-mechatronic technology in the fields of precision manufacturing and integration that the temperature control system can construct a stable and controllable temperature field within a limited space, achieving simultaneous improvement in heat transfer efficiency and regulation accuracy.

## Figures and Tables

**Table 1:** Comparison of key thermal performance indicators.

Instrument Type	Up Ramp (°C/s)	Down Ramp (°C/s)	Steady-State Temperature Deviation (±°C)	Single Cycle Duration (s)	Total Duration for 35 Cycles (min)
Liquid metal bath	1.2 ± 0.1	0.8 ± 0.1	0.3	82 ± 3	47.8 ± 1.2
Peltier-cooled air bath	1.5 ± 0.1	1.1 ± 0.1	0.4	75 ± 2	43.8 ± 0.8
Air bath	7.5 ± 0.1	13.5 ± 0.1	0.1	28 ± 2	16.3 ± 0.6

**Table 2:** Thermal performance differences among various configurations.

Structural Configuration	Temperature Uniformity Inside Heating Chamber (°C)	Time Taken for Heating Up to 95 °C (s)	Time Taken for Cooling Down to 55 °C (s)	Sample Amplification Efficiency After 35 Cycles (%)
Scheme A (Equal height short cylinder)	1.2	17.3 ± 2	11 ± 1	92.5 ± 1.3
Scheme B (Unequal height)	1.8	16 ± 2	10.3 ± 1	88.3 ± 1.5
Scheme C (Equal height long cylinder)	0.1	14 ± 1	9 ± 1	98.9 ± 0.2

## Data Availability

The raw data supporting the conclusions of this article will be made available by the authors on request.
